# Tip-Based Nanofabrication for Scalable Manufacturing

**DOI:** 10.3390/mi8030090

**Published:** 2017-03-16

**Authors:** Huan Hu, Hoe Joon Kim, Suhas Somnath

**Affiliations:** 1 Department of Science and Solutions, IBM T.J. Watson Research Center, Yorktown Heights, NY 10598, USA; 2 Department of Robotics Engineering, Daegu Gyeongbuk Institute of Science and Technology (DGIST), Daegu 42988, Korea; kim.hoejoon@gmail.com; 3 The Center for Nanophase Materials Sciences and The Institute for Functional Imaging of Materials, Oak Ridge National Laboratory, Oak Ridge, TN 37831, USA; suhas.somnath@gmail.com

**Keywords:** tip-based nanofabrication, scanning probe lithography, scalable nanomanufacturing, atomic force microscope, scanning tunneling microscope

## Abstract

Tip-based nanofabrication (TBN) is a family of emerging nanofabrication techniques that use a nanometer scale tip to fabricate nanostructures. In this review, we first introduce the history of the TBN and the technology development. We then briefly review various TBN techniques that use different physical or chemical mechanisms to fabricate features and discuss some of the state-of-the-art techniques. Subsequently, we focus on those TBN methods that have demonstrated potential to scale up the manufacturing throughput. Finally, we discuss several research directions that are essential for making TBN a scalable nano-manufacturing technology.

## 1. Introduction

Since its inception, the ability to fabricate structures and devices at the nanoscale has been the cornerstone of nanotechnology. Nanofabrication can generally be categorized into top-down and bottom-up approaches, although some emerging fabrication techniques are a combination of the two approaches. Among the two approaches, top-down nanofabrication has been driving the massive success of semiconductor industry for more than 50 years. Industrial applications mostly use optical lithography that uses photons to pattern photoresist. The patterned photoresist reveal the underlying sample that facilitates the etching of the exposed material to transfer these nanopatterns onto the desired materials. The photo-masks used in lithography are mostly prepared using electron beam lithography [1], and the state-of-the-art masks can have a spatial resolution smaller than 10 nm [2,3]. These top-down approaches require high capital and operational costs, are applicable only to planar surfaces, and are incompatible with biological materials [4]. Bottom-up approaches such as soft lithography [5,6] and self-assembly [7] are relatively inexpensive and offer scalability, but suffer from limited design geometries and lack of control of the shape and position of nanostructures. Hybrid nanofabrication approaches such as nanoimprint lithography [8,9], edge lithography [10,11], and stencil lithography [12,13] are promising in terms of reducing fabrication costs and offering more flexibility in design of nanopattern geometries. Other nanofabrication methods such as laser interference lithography [14,15,16] and maskless etching [17,18,19] are capable of patterning large areas with specific patterns, but are not suitable for patterning arbitrary shapes.

Tip-based nanofabrication (TBN) is a rapidly growing alternative to photolithography in the top-down nanomanufacturing category. TBN uses a physical probe in contact or in close proximity to a surface to modify the surface using thermal, mechanical, or electrical fields. Furthermore, some TBN techniques have the potential to become truly controlled approaches for manufacturing nanostructures and nanoscale devices for the upcoming decades [20].

In this review paper, we will introduce the history and the technology development of TBN in the first section. In the second section, we will briefly review various TBN techniques that use different physical or chemical mechanisms to fabricate features and discuss some of the state-of-the-art techniques. In the third section, we will review those TBN methods that have demonstrated potential for fabricating nanoscale devices and have the potential for scaling up the manufacturing throughput. In the last section, we will discuss several research directions that are essential for making TBN a scalable nano-manufacturing technology.

## 2. History of Tip-Based Nanofabrication

TBN can trace its history back to 1981, when two international business machines (IBM) researchers—Gerd Binnig and Heinrich Rohrer invented the scanning tunneling microscope (STM), a device capable of measuring the topography of a conductive sample by tracking the tunneling current from an atomically sharp conical probe to the sample. Ever since, STMs have spearheaded a new field for the study of the morphology of matter at the atomic scale [21]. Shortly afterwards, Binnig invented the atomic force microscope (AFM) in 1986. AFMs used probes consisting of a microcantilever beam with an atomically sharp tip at the free-end of the beam in close proximity or in contact with the sample surface. The properties of the sample are measured by tracking the bending motion of the compliant cantilever beam as the tip scans the sample by shining a laser beam off the back of the cantilever. Unlike STMs, AFMs are capable of imaging a variety of samples including conductive, non-conductive [22], and even biological samples [23,24]. Today, STMs and AFMs are the most popular examples of scanning probe microscopes (SPMs).

Besides atomic resolution imaging, the unprecedented resolution of the STM also facilitated the observation of adsorbed atoms and molecules, which enabled the possibility of surface modification using the STM. Since the 1990s, researchers have demonstrated various STM nanofabrication applications such as locally pinning molecules to surfaces [25], transferring atoms from the tip to the surface [26], positioning single xenon atoms on a nickel surface [27], and removing hydrogen atoms from a silicon surface [28]. In 2000, IBM used a 32 × 32 array of individually addressable thermal AFM cantilevers for thermally writing, imaging, and erasing bits for a data storage device called the “Millipede” [29,30]. As the instrumentation and probes continued to develop, researchers have developed many more novel nanofabrication applications using SPMs. The next section introduces different mechanisms for fabricating nanostructures using tips.

## 3. Overview of Tip-Based Nanofabrication (TBN)

**Figure 1:**
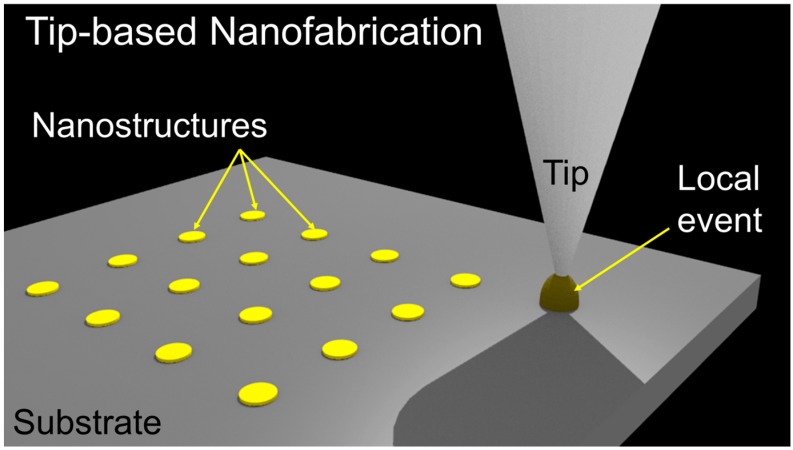
A cartoon illustration of tip-based nanofabrication. A tip is brought into close proximity with a substrate. An external stimulus to the tip results in a physical or chemical event localized to the tip-surface junction resulting into the fabrication of a nanostructure. The tip is then moved to the next position to fabricate the next structure.

In TBN, a nanoscale tip is brought into close proximity or contact with a substrate as shown in Figure 1. This is typically achieved using a feedback loop with a way of measuring the distance between the tip and the substrate and a stage of precisely moving the tip away or close to the substrate. Once the tip is maintained at a specific distance from the substrate, an external stimulus is applied to the tip to initiate certain events such as joule-heating within the tip, bias induced electro-chemical reaction, force induced mechanical indentation, or molecular diffusion, localized to a nanoscale volume between the tip and the substrate. Once this event completes, the tip is moved to another location by an *X*-*Y*-*Z* stage to fabricate next nanopattern.

TBN techniques can be categorized by the instrumentation platform to be STM-based TBN and AFM-based TBN. TBN can also be categorized by the underlying mechanisms that enable the fabrication such as atom-removing, thermal, electro-chemical, optical, molecular diffusion, mechanical removal, and field emission. Some TBN techniques are facilitated by a combination of two or more mechanisms such as thermo-mechanical TBN. We will review TBN techniques in each mechanism in the following sections.

### 3.1. Atom Removing-Based TBN

This type of TBN can trace back to the demonstration of single atoms positioning on a metallic surface conducted by two IBM researchers Eigler and Schweizer in 1990 [27]. They used a low-temperature ultra-high vacuum (UHV) STM system. They pulled Xenon atoms across a surface while the xenon atoms remained bounded to the surface. Shortly after this, Lyding at the University of Illinois as well as researchers at IBM demonstrated that STM tips can also be used to remove hydrogen (H) atoms from a silicon (100) 2 × 1 surface [28,31] in UHV. On a (100) 2 × 1 silicon surface, there is a single unsatisfied covalent bond, also called a dangling bond, that can be passivated with a single hydrogen atom. STMs can achieve atomic-resolution patterning by removing a single hydrogen atom from a Si (100):H surface.

**Figure 2:**
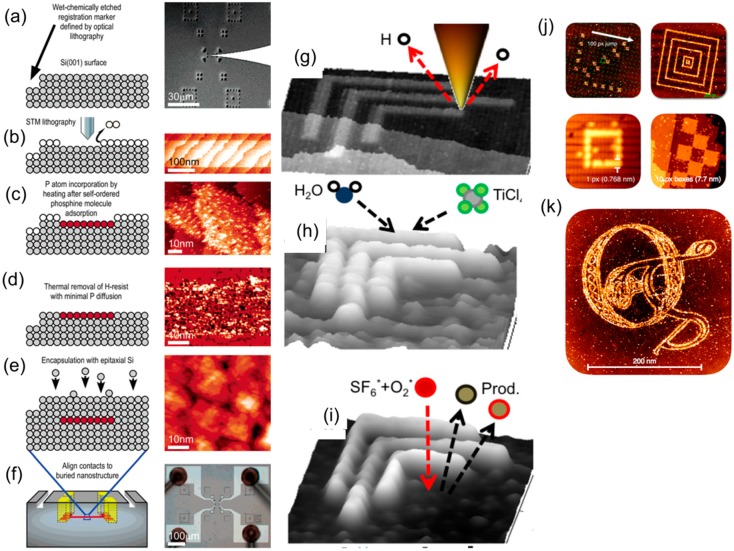
(**a**–**f**) Fabrication process of a nanoelectronic devices using hydrogen depassivation lithography (HDL) [37]: (**a**) surface markers are created prior to HDL for alignment purpose; (**b**) the STM tip is used to pattern lines around 10 nm on the Si(100):H surface; (**c**) PH_3_ selectively adsorbs to the patterned region and forms a phosphorus layer after heating; (**d**,**e**) the hydrogen resist is removed and phosphorous atoms are buried after the epitaxial growth of silicon; (**f**) ex situ fabrication of aluminum contact electrodes to the embedded devices for electronic testing occurs. (**g**–**i**) Schematic illustration of the fabrication process of silicon nanostructures using HDL [35]: (**g**) STM tip selectively removes regions of a monolayer of H atoms to make it chemically active; (**h**) after exposure to atmosphere and partial oxidation, atomic layer deposition of H_2_O+TiCl_4_ is used to form a 2.8 nm thick hard mask of TiO_2_; (**i**) reactive ion etching is used to etch 17 nm deep silicon with TiO_2_ as etch mask. (**j**,**k**) Arbitrary shapes of nanopatterns created by ZyVector tool based on HDL, figures reprinted with permission from ZyVex Labs.

Hersam et al. studied the chemical robustness of a Si(100):H surface using X-ray photoelectron spectroscopy (XPS) and found no evidence of oxidation until after 40 h of exposure in ambient conditions [32]. The superior passivation uniformity of Si(100):H surfaces in comparison to Si (111) surfaces has caused the former to be used far more widely in the semiconductor industry [33]. Continued development of this patterning technique has led to the mature technique known today as hydrogen depassivation lithography (HDL) [34,35,36]. Ruess et al. used this technique to fabricate devices made of phosphorous atoms that were placed instead of the hydrogen atoms that were removed from the silicon surface [37]. Zyvex Labs has also demonstrated pattern transfer techniques using patterned hydrogen atoms as templates for selective Si and Ge epitaxial growth [38], the atomic layer deposition (ALD) of TiO_2_ [35], and the three-dimensional growth of nanostructures [39]. Unlike many TBN techniques, this TBN technique has the advantage of a sub-nm resolution and has the potential to fabricate prototypes of infinitesimally small devices such as atomic scale devices [37]. Figure 2 illustrates the fabrication process of (a–f) a nanoelectronic device, (g–i) silicon nanostructures, and (j–k) arbitrarily shaped nanopatterns using HDL. Currently, HDL is still limited by its slow speed (typically 80 nm/s [40]) and the requirement of UHV and low-temperature environment. However, considering the superior resolution of HDL and the continued trends of shrinking device dimensions in the semiconductor industry, motivations are still strong to overcome technical challenges and to increase the commercial applicability of HDL.

### 3.2. Thermal TBN Techniques

Thermal TBN techniques typically use a probe capable of self-heating to fabricate nanostructures. The heated probe can initiate nanofabrication via either additive or subtractive methods. Heated AFM probes were initially developed for data-storage applications in the early 1990s [41,42,43,44]. Since then, they have also been employed for thermal analysis [45,46,47,48,49], thermal-imaging [50,51], displacement sensing [52], and nanofabrication purposes [53,54]. These heated probes are typically made of differentially doped silicon such that a passing current through the probe causes joule heating at the heater region that is close to the atomically sharp tip. Such probes allow for a control of temperature and heat flow at the nanometer scale, thereby enabling thermal processing techniques and applications at the nanometer scale [55].

**Figure 3:**
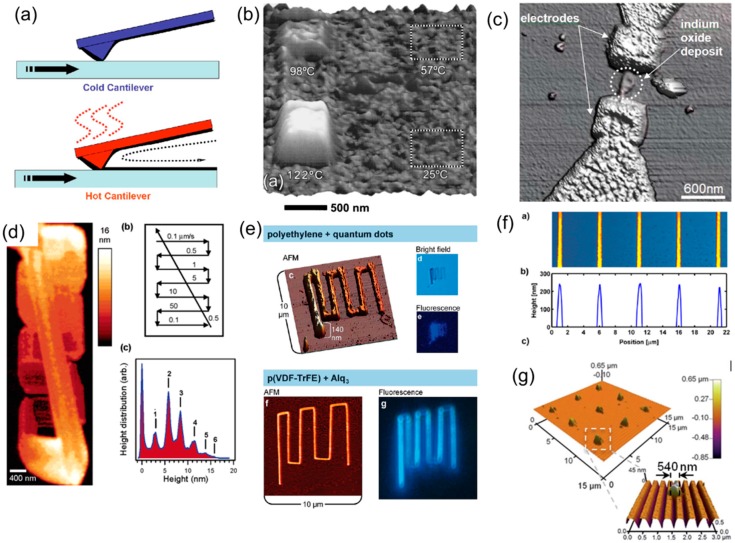
(**a**) Schematic representation of depositing inks using a heated atomic force microscope (AFM) tip [69]; (**b**) AFM topography image of octadecylphosphonic acid (OPA) nanopatterns deposited by scanning the heated AFM tip at different temperature at four 500 nm × 500 nm square areas [56]; (**c**) indium oxide nanolines deposited by a heated AFM tip bridging two prefabricated electrodes [56]; (**d**) conductive polymer oly(3-dodecylthiophene) PDDT nanostructures deposited by a heated AFM tip on a silicon oxide substrate [57]; (**e**) polyethylene mixed with quantum dots nanopatterns as well as nanopatterns of Alq3 deposited using a heated AFM tip [59]; (**f**) array of polyethylene nanolines with 3 μm gap and 400 nm width deposited by a heated AFM tip [62]; (**g**) 3 × 3 nanodot array of polystyrene with 500 nm size deposited on a photonic crystal surface [61].

Sheehan and King demonstrated an additive thermal TBN technique called thermal dip pen nanolithography (tDPN) in 2004, where a heated probe deposited indium, a metal with a relatively low melting temperature, on a substrate [56]. Later, tDPN was expanded to additional classes of materials such as conductive polymers [57], stimulus-responsive polymer [58], nanoscale polymer composites with nanoparticles [59], and heterogeneous polymer nanostructures [60]. Thermal probes can also deposit nanoscale polymer patterns on a non-flat surfaces such as a photonic crystal [61]. Figure 3a shows the schematic illustration of the working principle. When the AFM tip is not heated, it does not deposit ink on a substrate. The solid ink melts and flows from the tip to the substrate only when the probe is heated above the melting temperature of the ink. Figure 3b shows the AFM topography image of octadecylphosphonic acid (OPA) nanopatterns deposited by scanning the heated AFM tip at different temperature at four 500 nm × 500 nm square areas [56]. Figure 3c shows indium oxide nanolines deposited by a heated AFM tip bridging two prefabricated electrodes [56]. Figure 3d shows conductive polymer oly(3-dodecylthiophene) PDDT nanostructures deposited by a heated AFM tip on a silicon oxide substrate [57]. Figure 3e shows nanopatterns of polyethylene mixed with quantum dots as well as nanopatterns of Alq3 deposited using a heated AFM tip [59]. Figure 3f shows an array of polyethylene nanolines with a 3 μm gap and a 400 nm width deposited by a heated AFM tip [62]. Figure 3g shows a 3 × 3 nanodot array of polystyrene with a 500 nm size deposited on a photonic crystal surface [61].

The feature size defined by this additive thermal TBN technology is dependent on the tip radius, the tip scan speed, and the mass flow rate of ink. The mass flow rate of ink is determined by the tip temperature, the polymer viscosity, and the substrate temperature. Once the mass flow rate is determined, the deposited line width will decrease as the tip scanning speed increases. Felts et al. studied the mechanisms of polymer flow of molten polyethylene during the deposition process by a heated AFM tip and concluded that the mass flow rate of the polymer was driven mostly by capillary force instead of substrate shear. Moreover, it was found that the mass flow rate was mostly dependent on the viscosity of the molten polymer [60].

**Figure 4:**
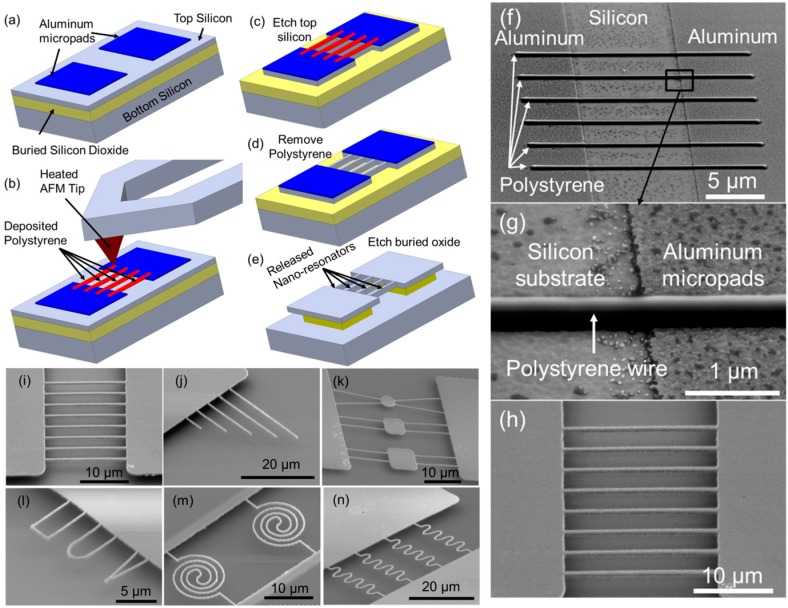
(**a**–**e**) Illustrations of the major steps of fabricating mechanical nano-resonators using tip-based nanofabrication (TBN) and conventional optical lithography: (**a**) Fabricate aluminum micropads on top of an silicon on insulator (SOI) wafer using conventional optical lithography and etching; (**b**) deposit polystyrene nanowires across the two aluminum micropads with a heated AFM tip; (**c**) etch top silicon using micropads and deposited polystyrene nanowires as the etch mask (this step creates silicon nanobeams); (**d**) remove polystyrene nanowires by acetone; (**e**) etch buried oxide to release suspended silicon beams. (**f**,**g**) Scanning electron microscope (SEM) images of deposited polystyrene nanowires across two aluminum micropads; (**h**) SEM image of suspended silicon nanobeams after etching buried silicon oxide. (**i**–**n**) Six different types of silicon mechanical nano-resonators fabricated using the process shown in (**a**–**e**).

While the working principle was well understood, much of the tDPN work was limited to drawing dots and lines of polymer inks. One of the biggest challenges in applying tDPN for fabricating working nanodevices was the integration of tDPN with established semiconductor manufacturing techniques. To solve the challenge, Hu et al. tested a vast library of polymer inks to determine if they meet the criteria for serving as an etch mask for integration into the semiconductor manufacturing process. First, the polymer needs to survive the harsh chemical and ion etching processes; second, the polymer should be removed selectively without contaminating the underlying and surrounding area on the substrate; third, the polymer needs to adhere well to the substrate and compatible with tDPN [63]. Moreover, Hu et al. demonstrated that these polymer nanopatterns can be combined with a microscale mask defined by optical lithography together to fabricate nanoelectromechanical system (NEMS) devices [64] such as mechanical nano-resonators [65], nanofluidic channels [66], and silicon transistors [67], as well as graphene nanoribbons for transistors [68] as seen in Figure 4. Figure 4a–e show a typical process for integrating a TBN approach with conventional optical lithography to fabricate NEMS mechanical nano-resonators. This TBN process is very robust, operates in ambient conditions, and is easy to implement. More importantly, this TBN process is compatible with conventional microfabrication process and therefore very suitable for nanodevice prototyping.

Heated AFM tips can also initiate nanometer scale thermal-chemical reactions on substrate, an approach known as thermal-chemical nanolithography (TCNL). This approach was firstly demonstrated in 2004 to induce photoresist cross-linking at the nanometer scale [70]. TCNL was also used to pattern diazide–dyine thin films by thermally induced cycloaddition [71]. In one example, TCNL was employed to convert a precursor polymer film to a widely used electroluminescent poly(p-phenylene vinylene) with sub-100 nm spatial resolutions [72,73]. In addition, TCNL was also used to reduce graphene oxide to conducting graphene nanoribbons [74].

Instead of initiating chemical reactions, heated AFM tips can also induce physical change such as sublimation [75] or physical decomposition of polymer [76]. Knoll’s group from IBM Zurich recently demonstrated sub-20 nm silicon patterning using a patterned polymer as an etch mask through a series of pattern transfers [77]. This approach requires a customized polymer resist with tailored properties to achieve optimal performance [78].

### 3.3. Electro-Chemical TBN

**Figure 5:**
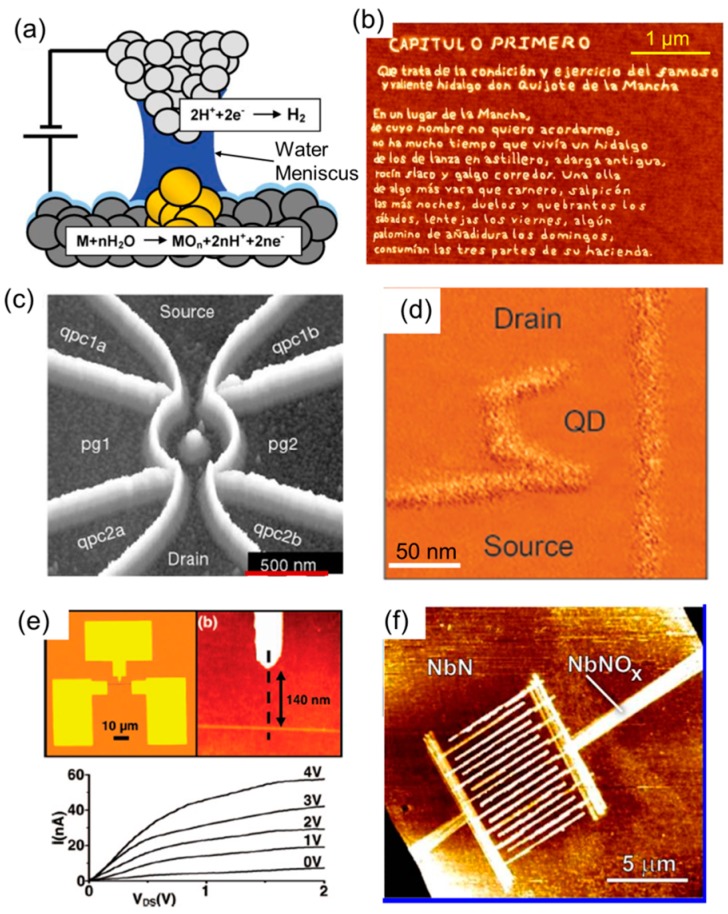
(**a**) Schematics illustrating local oxidation of a metal surface. (**b**) First paragraph of “Don Quixode” defined by LON. (**c**) AFM image of a quantum ring fabricated by LON. (**d**) Quantum dot defined by local oxidation. (**e**) Silicon nanowire transistor fabricated by LON and the drain current versus drain voltage at different gate voltage setting. (**f**) Superconducting 4 nm thick niobium nitride (NbN) nanowires defined by local anodization.

Electro-chemical TBN techniques apply an electric bias between the tip and the substrate to induce local electrochemical reactions to fabricate nanopatterns. The electrochemical reaction can be the chemical change of materials such as inducing anodic oxidation of semiconductors [79] and metals [80], or the deposition of materials from the environment. One robust approach that has demonstrated the capability of device fabrication is local oxidation nanolithography (LON). Following the discovery of oxidation of passivated silicon by tip in an STM system in 1990 [79], Day and Allee demonstrated the oxidation of silicon with sub-100 nm resolution using a tip in an AFM system. The use of an AFM instead of an STM obviated the need for a vacuum environment, thereby making LON easier to implement [81]. Figure 5a shows the typical setting of LON for an oxidizing metal surface. The water meniscus (as small as 20 nm) that forms when an AFM tip is brought close to a metal substrate, serves as the electrolyte when a negative electric bias is applied between the AFM tip (cathode) and the substrate (anode). Thus, arbitrary shapes of oxide features can be fabricated, such as a paragraph of “Don Quixote”, as shown in Figure 5b. LON has been used to fabricate many types of nanoscale devices such as quantum devices [82,83,84], nanowire field-effect transistors [85], graphene devices [86,87,88], MoS_2_ transistors [89], photonic devices [90,91] and silicon microlenes [92]. Figure 5c shows an AFM image of a quantum ring fabricated using LON. Figure 5d shows a quantum dot defined by local oxidation. Figure 5e shows a silicon nanowire transistor fabricated using LON and the corresponding drain current versus drain voltage at different gate voltages. Figure 5f shows 4 nm thick superconducting niobium nitride (NbN) nanowires fabricated by using LON. The resolution of LON is affected by many parameters such as the relative humidity of environment, the applied voltage amplitude, the duration of applied electric bias, the scanning speed, and the tip-substrate distance. Researchers have demonstrated the fabrication of silicon oxide nanostructures with a lateral size ranging from 10 to 100 nm and a height in the range of 1–10 nm using LON. The upper limit of the scanning speed is set by the required time for the chemical reaction to occur, which falls in the range of 0.5 µm/s to 1 mm/s.

### 3.4. TBN-Using Optics

**Figure 6:**
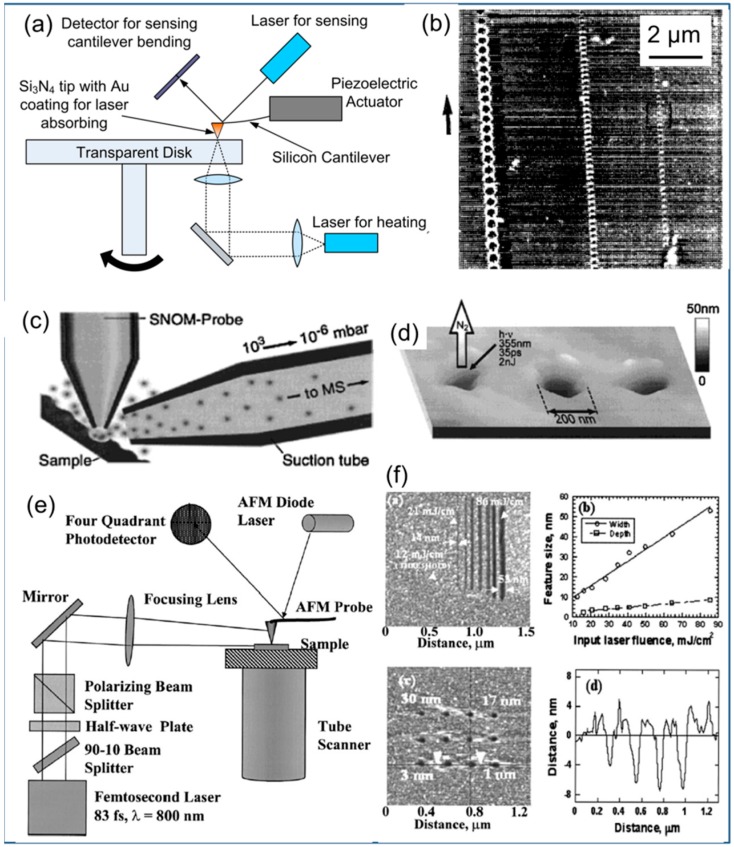
(**a**) Experiment setup for thermal-mechanical writing with an AFM tip on a rotating sample. A focused laser beam propagates through a transparent polymethylmethacrylate (PMMA) film and heats the optically absorbing AFM tip, which in-turn softens the PMMA film indents the film. (**b**) AFM topography image of tracks written at 50–100 KHz using method shown in (**a**) [41]. (**c**) Schematics showing the near-field laser ablation/nanosample interface for mass spectroscopy. A laser propagates through the fiber tip and ablates a nanoscale region of the plume sample [96]. (**d**) Topography image of a triazene sample surface following the ablation experiment [96]. (**e**) Schematic showing the experiment setup for field enhanced fabrication. A femtosecond laser is incident on AFM tip and create enhanced electrical field, which enables the nanofabrication [103]. (**f**) The dependence of lateral feature sizes on laser fluence and tip-sample separation distance. Features as small as 10 nm are fabricated [103].

TBN-using optics can be divided into three main categories. The first uses the thermal effects of light. IBM showed that a laser could be used to heat an SPM tip, which softened the thin polymer film in contact with the tip and left an indentation on the polymer film [41] as shown in Figure 6a. Here, the laser was incident on the back of the tip and there were no near field effects. Though the laser spot was substantially larger, the nanometer size is due to the sharpness of the tip. Figure 6b shows AFM images of 150 nm wide indentations with a 250 nm pitch made by the tip using this TBN method. The feature resolution for this method is mostly defined by the tip geometry and how deep the tip indents the polymer film.

The second method takes advantage of the working principle of the near field scanning optical microscope (NSOM). Light is delivered to the substrate using an optical fiber tip to expose conventional photoresists [93], photosensitive polymers [94] or to modify ferroelectric substrates. Using the NSOM configuration, a laser could be used to ablate materials such as thin organic films [95,96] and metals. Figure 6c shows the typical setup for laser ablation through an optical fiber tip, and Figure 6d shows the nanostructures formed by ablation. The feature resolution in this technology is mostly dependent on the confinement of light delivered by the tip and is typically around 100 nm for a laser source with a wavelength of 543 nm [94].

The third optical TBN method is based on field enhancement induced by the tip. This type of TBN work dates back to the 1990s, when nanoscale tips were illuminated with nanoseconds of laser pulses to fabricate small hillocks with silver tips on a gold film in vacuum [97], nanodots with 20–30 nm size on Ge surfaces [98], and 10 nm wide nanopatterns on metal surfaces [99]. Recently, Xu’s group at Purdue University nanoscale bowtie optical apertures to focus laser spots in order to expose a photoresist film to create features with sub-100 nm dimensions in parallel [100]. A research group at the University of California at Berkeley demonstrated nanofabrication with 10 nm resolution using a femtosecond laser pulses onto an AFM tip [101]. Recently, the same group grew a single nanowire by confining a laser spot laser onto a gold nanoparticle while applying electrical bias through the tip [102]. Figure 6e shows the experimental setup where a femtosecond laser beam is focused on the AFM tip to create a tip-enhanced electric field to create nanostructures. Figure 6f shows the dependence of lateral feature sizes on the laser fluence and the tip-sample distance. In this type of TBN method, the resolution is determined by the laser flux and tip-sample distance. Feature dimensions increase almost linearly with energy flux of the laser pulse. The high repetition rates of the femtosecond laser system ensure a fast nanofabrication rate. The strong spatial field confinement at the tip-sample interface makes the scattering effects from the multi-tip array insignificant, which is very promising characteristic for scaling the technique up to become a high-throughput nanofabrication technology [103].

### 3.5. TBN Using Molecular Diffusion

**Figure 7:**
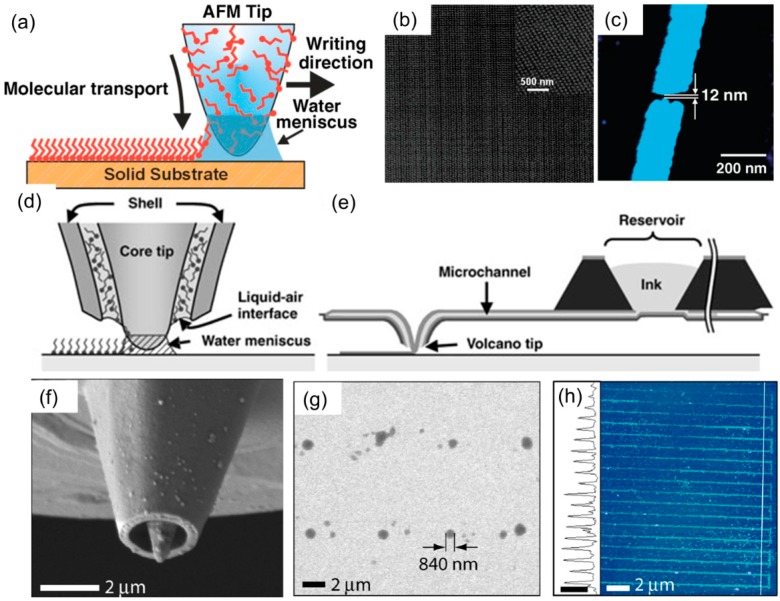
(**a**) Schematic representation of the dip pen nanolithography (DPN) process. A water meniscus forms in between tip and substrate which facilitates molecular transport from the tip to substrate [105]. (**b**) Tapping mode AFM images of 60 nm gold nanodots deposited by DPN and subsequent etching [111]. (**c**) 12 nm gold nanogap fabricated by DPN and subsequent etching [110]. (**d**) Schematic representation of fountain pen nanolithography. The ink is dispensed through the hollow tip to the substrate [112]. (**e**) Schematic representation of the nanofountain pen probe structure. A micro reservoir for storing inks is connected to the volcano tip through a microfluidic channel [112]. (**f**) SEM image of the volcano tip [116]. (**g**) SEM image of a 2 × 4 array of anti-BSA IgG dots patterned on a BSA substrate (46% RH) by fountain pen [116]. (**h**) Tapping-mode AFM image and height profile of parallel lines of biotin-BSA patterned on 6-Mercaptohexanoic acid (MHA) at a translation rate of 80 µm/s (50% RH, height scale bar in profile is 20 nm) [116].

In 1995, German scientists reported the deposition of octadecanethiols (ODT) using an AFM tip on a mica surface [104]. Later in 1999, Mirkin’s group deposited alkanethiols on a gold surface with 30 nm line-width resolution using an AFM tip, marking the invention of dip pen nanolithography (DPN) [105]. The basic concept of DPN is shown in Figure 7a. When an AFM tip is close to a solid substrate, a water meniscus forms due to humidity in the environment, and the molecules on the surface of the AFM tip can diffuse into the substrate through this water meniscus. This is a direct write technique that can deposit chemicals and biological samples to the substrate. Since its inception, there have been several review papers specifically focusing on DPN [106,107]. Tip-substrate molecular transport is a complicated process and influenced by many parameters such as the tip shape, surface chemistry, motility of ink on the tip, temperature, humidity of environment, water solubility of the ink, etc. De Yoreo et al. studied the deposition of 6-Mercaptohexanoic acid (MHA) on gold substrates and observed an increase in deposition rate with increasing humidity [108]. Sheehan and Whiteman conducted a similar study on ODT and found a negligible dependence of the deposition rate on the humidity [109]. The different behaviors of ODT and MHA can be explained in terms of their differences of solubility in the water meniscus [107]. The transport process in DPN is typically slow and is limited by the mass transport of molecules. Typically, the dwelling time for fabricating a nanodot of molecules ranges from hundreds of milliseconds to seconds. Given a spot size of 50 nm and a 100 ms dwelling time, the scan speed of DPN is estimated to be around 500 nm/s. Figure 7b,c show sub-50-nm gold nanodot structures as well as a 12 nm wide gap in a gold nanoline [110]. Nanopatterned chemicals deposited by DPN served as an etch mask for subsequent etching of gold [110,111].

The fountain pen design principle was used to develop a new DPN probe to enhance the capabilities of DPN from ink-and-write to writing via continuous ink delivery [112] As shown in Figure 7d,e, the ink was dispensed through an aperture inside the tip from a microfluidic reservoir. Figure 7f shows a scanning electron microscope (SEM) image of the fountain pen tip. Similar to DPN, the size of features fabricated using fountain pen tips is dependent on many parameters including the size of the water meniscus which can be as small as 10 nm, the solubility of ink molecules in water, the tip velocity which determines the duration that the tip stays at each spot, and the surface chemistry of the substrate.

Researchers have used fountain pen tips to deposit DNA strands [113], gold nanoparticles [114], pattern patterning cells, and inject nanoscale diamonds into single-cells [115]. Figure 7g shows a 2 × 4 array of anti-bovine serum albumin (anti-BSA) IgG dots patterned on a BSA substrate (46% RH) by fountain pen tips [116]. Figure 7h shows an AFM image and height profile of parallel lines of biotin-BSA patterned on MHA at a tip-translation rate of 80 µm/s [116].

### 3.6. TBN via Mechanical Removal

TBN via mechanical removal represents a miniaturization and direct mimicking of macro-scale machining technologies. Early works were demonstrated in STM systems and were limited to metals and graphite surfaces. For example, features 30 nm in diameter and 10 nm in depth were produced by an STM tip operating at a low tunneling resistance below 85 MΩ on a thin sputtered polycrystalline gold film [117]. Similarly, 70 nm wide and 23 nm deep grooves have been etched into a thin gold film on a mica substrate using a tungsten STM tip [118].

**Figure 8:**
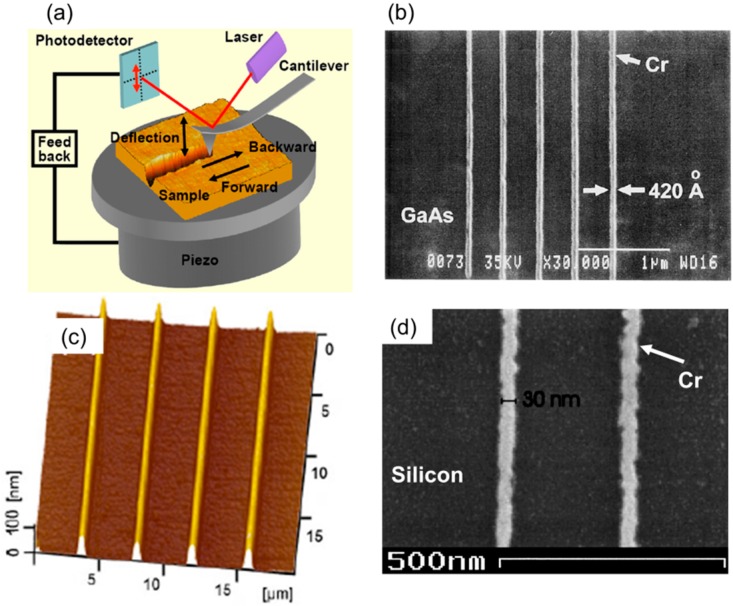
(**a**) Schematic representation of mechanical removal process using an AFM tip in a typical AFM system [119]. (**b**) 42 nm wide Cr line arrays on top of a GaAs substrate fabricated by mechanical removal TBN process [144]. (**c**) Protruded silicon nanoline structures on top of a silicon substrate fabricated by mechanical TBN and subsequent etching [138]. (**d**) 30 nm wide Cr line arrays on top of a silicon substrate fabricated by mechanical TBN and subsequent etching and metal lift off process [133].

One popular method in this TBN category uses a tip to directly remove substrate material as shown in Figure 8a [119]. While this method is straightforward and easy to implement, it suffers from several issues such as rough edges, tip wear, and bad feature quality. Hard materials such as solid diamond tips [120,121,122,123], diamond-coated tips [124], and carbon-coated tips [125] have been used to reduce tip wear. Instead of operating AFM tips in contact mode, operating AFM tips in tapping mode to plow surfaces (dynamic plowing) [126,127,128,129,130,131] can also improve consistency in the fabricated features. The precision of the fabricated features has been improved by integrating mechanical removal with other approaches such as subsequent etching [132] or by introducing an intermediate resist layer [133] are demonstrated [133,134,135,136,137,138]. Recently, Yan et al. demonstrated three-dimensional nanostructures with 100 nm lateral resolution [139,140,141,142] using these techniques.

Unlike conventional machining process in which the shape of the fabricated features is defined by the relative motion between the tool and the work-piece, this mechanical TBN method relies on the normal force control during machining. The machined depth is determined by the normal force, the tip geometry, and the mechanical properties of samples [139]. The lateral resolution of nanopatterns generated by this mechanical TBN method depends on the mechanical property of the substrate material, the applied force magnitude, the tip geometry, and the magnitude of the applied force on the tip. The scan speed of the AFM tip is usually not fast (0.1–10 µm/s), since fast tip scan speeds (e.g., 100 µm/s) can fracture the tip [119]. Wear rate is crucial for this mechanical TBN method and is highly dependent on the mechanical properties of substrate and the tip. For example, a typical diamond-like-carbon (DLC) coated tip wears at 2.626 × 10^−7^ mm^3^/(N·m) on a 20 nm thick Pt film produced [119], and increases to 2 × 10^−6^ mm^3^/(N·m) on more rigid surfaces like a silicon oxide film [143].

Overall, this mechanical TBN technology is inexpensive, versatile, easy to implement, and is suitable for prototyping nanostructures. Nonetheless, there are several technical challenges such as tip wear and inconsistency in the dimensions of fabricated nanopatterns to overcome before it develops into a reliable nano-manufacturing technology.

### 3.7. TBN Using Field Emission

**Figure 9:**
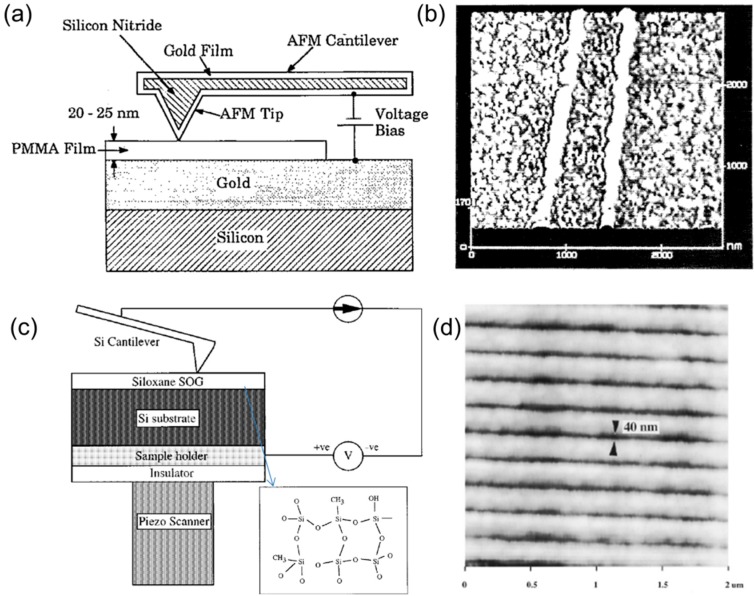
(**a**) Experiment setup for chemically modifying a PMMA film using a gold-coated silicon nitride AFM tip. A voltage bias is applied across the 20–25 nm thick PMMA film between the metal-coated tip and the underlying gold film [149]. (**b**) AFM image of two 100 nm wide protruding lines created by scanning the metal-coated AFM tip under a −18 V bias. [149]. (**c**) Schematic diagram of experiment setup for modification of siloxane spin on glass (SOG) film. A voltage bias is applied across the 100 nm thick SOG film between the silicon tip and underlying substrate. The inset shows the siloxane structure. Si–CH_3_ (siloxane) and Si–OH (silanol) are present [150]. (**d**) SOG Nanolines of 40 nm width and 200 nm pitch written with 0.8 nA current and a tip scan speed of 180 µm/s. [150].

Early examples of TBN using field emission of electrons were demonstrated in vacuum using an STM platform. An STM tip’s ability to confine the low energy electron beam overcomes the degradation in resolution due to secondary and backscattered electrons produced by high energy electron beams in conventional e-beam lithography systems [145]. Twenty nanometers of resolution in positive resist [146] and 30 nm of resolution in negative resist were demonstrated using this technique [147]. The quality of the patterns fabricated was found to depend on the applied bias, the resist film thickness, and the substrate surface roughness [148]. Shortly after the initial demonstration in STM systems, TBN using field emission was also demonstrated in AFM systems in ambient conditions instead of in vacuum. In 1992, Majumdar et al. patterned 35 nm wide polymethylmethacrylate (PMMA) resists using a localized electron source [149]. Figure 9a shows the experimental setup for this TBN method [149]. A gold-coated AFM tip made of silicon nitride was brought into contact with a Au/Si substrate coated with a 20–25 nm thick PMMA resist film. A voltage applied between the tip and the gold substrate induced electrons to shoot from the tip to the substrate, thereby exposing the PMMA film. Figure 9b shows an AFM topography image of two protruding, 100 nm wide lines in the PMMA film that were fabricated by scanning the tip with a −18 V bias [149]. In addition to the polymer resist, spin-on glass was also patterned in AFM systems with 40 nm resolution [150]. The low-voltage electron beams used in this technique do not suffer from proximity effects unlike high voltage electron beams. Furthermore, expensive and complicated electron optics are not needed to focus the beam. The resolution of the feature fabricated using this TBN method is determined by the magnitude of the applied electric bias between the tip and sample, the tip radius, the tip scan speed [150], and the resist thickness. The lateral resolution increases with increasing electric bias and decreasing tip scan speed. The reported tip scan speed ranges from 1 µm/s [151] to 1 mm/s [150].

Table 1 summarizes various TBN techniques discussed above, including their reported feature resolution, tip scan speed (determining the throughput), material compatibility, environment requirement, their advantages, and their disadvantages.

## 4. Advancement of Scalable TBN Approaches

Scalability is a key factor that demines if a nanofabrication approach can turn into a massive manufacturing approach. For example, optical lithography is a successful approach in the large-scale fabrication of nanoscale features in semiconductor manufacturing because it is scalable. For example, a 300 mm thick wafer can be routinely patterned with nanoscale features using deep ultraviolet (UV) optical lithography. In this section, we will specifically look at TBN approaches that have demonstrated the potential for scaling up to become manufacturing technology. Scalable TBN here refers to TBN approaches that employ multiple tips to fabricate nanoscale features in parallel. Such methods generally use two kinds of tip arrays—arrays of passive tips and arrays of active tips. Passive tip arrays are incapable of addressing tips individually; consequently, all the tips in the array fabricate identical features. Conversely, active tip arrays can address tips independently and are capable of switching the fabrication on or off eat each tip.

### 4.1. Passive Tip Array

**Figure 10:**
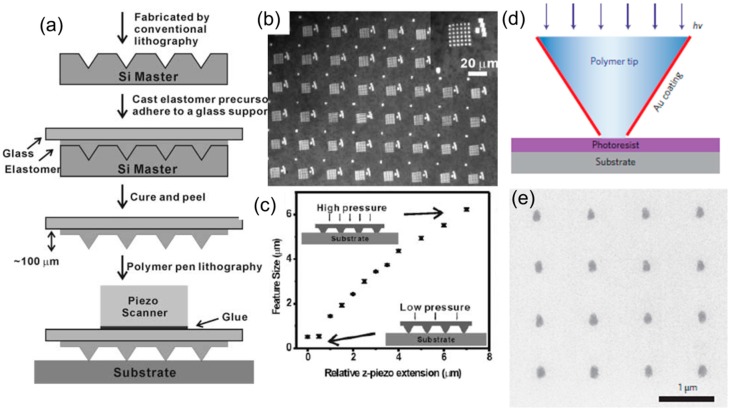
(**a**) Schematic representation of the polymer pen lithography experiment setup. Silicon mold is created using conventional optical lithography and anisotropic etching, which is used later as a mold to replicate polydimethylsiloxane (PDMS) pyramid tip array. PDMS tip array is glued to a glass substrate and attached to a piezo scanner to deposit inks on substrates. (**b**) Optical image of a 480 µm × 360 µm region containing one million gold dot array on a silicon substrate using polymer pen lithography (PPL). (**c**) MHA dot size as a function of *z*-piezo extension. (**d**) Schematic representation of beam pen lithography (BPL). Ultraviolet (UV) light is incident from the base of the transparent PDMS tip and goes through a sub-wavelength nano-aperture to expose photoresist in a region smaller than the wavelength of the light. (**e**) SEM image of chromium nanodots created by BPL followed with metal deposition and lift off.

One of the most important passive tip arrays was employed in the dip pen nanolithography (DPN). Tip arrays have been made using various types of materials, such as metal, silicon, silicon nitride, and polydimethylsiloxane (PDMS). Early work on DPN used an array of 32 silicon nitride tips [154], and later scaled up to an array of 55,000 tips [160]. “Polymer pen lithography” (PPL) used arrays of millions of polymer tips to fabricate features simultaneously where the feature size was modulated by the applied mechanical pressure, [161], and similar concepts were also demonstrated by Zhou’s group [162]. PPL has enabled many promising applications such as molecular printing at large scales and multiplexed inking for protein arrays [163,164]. Figure 10a shows the typical fabrication process for manufacturing PDMS tips used in PPL. Conventional optical micro-lithography is used to define silicon-etching windows, with a low-cost mask costing only a few hundred dollars. Due to unique etching characteristics of (100) single-crystal silicon, inverted pyramid shapes of trenches with nanometer-scale sharp apex can be formed, which later serve as molds to replicate PDMS tip arrays. Since the fabrication process is cheap and scalable, it is therefore easy to manufacture arrays with millions of tips. Figure 10b shows an optical microscopic image of a 480 µm × 360 µm region containing a million gold dots fabricated by PPL. Figure 10c shows the dependence of the pattern size on the contact pressure between the tip and the substrate, which was controlled by a piezoelectric actuator. Because soft PDMS tips can deform and result in a different contact area with the substrate if the contacting force is different, PPL was further optimized to use hard silicon nitride tips instead of soft PDMS tips [165] or to use a hard silica film coating PDMS tip array to improve the feature fidelity upon force variations [166].

Shortly after the invention of PPL, these PDMS tip arrays were used in a newly invented technology named beam pen lithography (BPL) to create patterns using UV light. Figure 10d shows the concept of BPL, where a PDMS tip is coated with an opaque metal coating except at the end of the tip. When UV light is incident from the base of the tip, light exposes a thin photoresist layer underneath the aperture. Because the physical size of the aperture is smaller than half the wavelength of the UV light, the near field effect makes it possible for the UV light to expose a region with less than half the wavelength of the UV light. Figure 10e shows the SEM image of a chromium nanodot array formed using BPL followed by a metal evaporation and lift off step. The size of the nanodot is about 111 nm in diameter.

**Figure 11:**
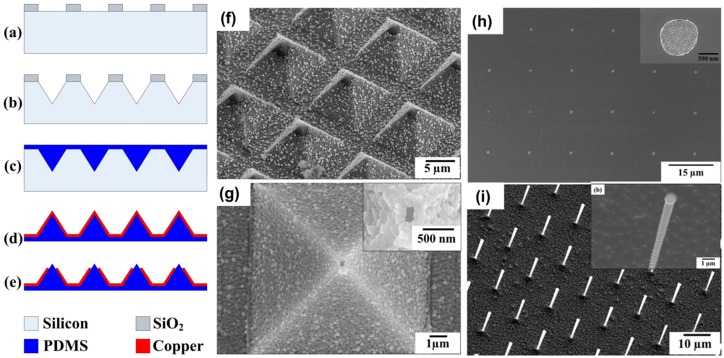
A scalable approach to fabricate massive numbers of nano-apertures: (**a**–**e**) process flow for fabricating nano-apertures using electrochemical etching; (**f**) SEM image of micro-aperture on PDMS tips fabricated by electrochemical etching for long time more than 20 min; (**g**) SEM image of nano-aperture on PDMS tip fabricated by electrochemical etching for only 2 min; (**h**) SEM image of aluminum nanodot array fabricated by exposure with nano-aperture array and subsequent metal deposition and lift off; (**i**) SEM image of silicon vertical nanopillar array fabricated by deep reactive ion etching of silicon using aluminum nanodot array as etching mask. The inset picture shows a magnified view of a silicon nanopillar.

However, the original paper reporting the invention of BPL does not have an effective method to fabricate massive amount of nano-apertures; instead, a slow and expensive focused ion beam process is employed to create the nano-apertures on the metal coating of PDMS pyramid tips, which significantly limits the scaling up of this BPL technique. Hu et al. in 2012 developed two approaches for mass-producing nano-apertures in a fast and scalable fashion [167]. One method is to use an electrochemical etching process to selectively remove the copper at the tip apex, and the other method is to use reactive ion etching to selectively remove the thin photoresist film at the tip apex, and further employ wet etching to form a chromium nano-aperture. Both methods are scalable and low-cost, and millions of nano-apertures with a size down to 100 nm can be fabricated in a very short time. Figure 11a–e shows the process flow of the electrochemical etching. Apertures with sizes from the micrometer down to 150 nm are prepared by adjusting the electrochemical etching duration. Figure 11f shows an array of micrometer-sized apertures etched for about 30 min. Figure 11g shows a nano-aperture with a size of about 150 nm using electrochemical etching. Figure 11h shows an array of 400 nm size aluminum dot defined by nano-aperture array. Figure 11i shows an array of vertical silicon nanopillars fabricated by deep reactive ion etching using the previously prepared aluminum dot arrays as the etch mask.

The same nano-aperture array was also used to define a gold dot array, which was then used to form Au seeds serving as catalysts for growing GaAs nanowires in well-positioned manner [168].

Liao et al. used similar methods to fabricate massive nano-aperture array and further demonstrated a desktop nanofabrication system with massively multiplexed light beams [169]. Several alternative approaches have been demonstrated to produce the nano-aperture arrays by spincoating carbon black photoresist [170], a combination of dry etching and electrochemical etching [171]. Zhou et al. demonstrated a technology called “apertureless-BPL” for scanning photochemical printing [172] based on the observation that transparent PDMS pyramids only allow light to come out from the tip based on the total internal reflection on the surfaces of the PDMS pyramids. Still, extra steps need to be taken to block light from passing through the space in between the pyramids by selective coating of the surface with metal. Wu et al. also demonstrated this apertureless-BPL based on a fully metal-coated polymer tip array, eliminating the step of selective coating metals [173]. Wu et al. also demonstrated a V-shaped PDMS tip coated with a thin film of metal for large-area sub-wavelength nanopatterning [174] as well as fabricating nanopatterns using the aperture existing at the oblique sidewalls of PDMS relief structures [175]. BPL is also used in a liquid form to synthesize nucleotides [176]. Liquid-phase BPL provides a way to perform local photochemical reactions that requires a liquid medium. Instead of doing resist exposure, molecules can be synthesized locally.

### 4.2. Active Tip Array

Tips that are thermally, electrically, chemically, or mechanically functionalized enable scalable fabrication and integration of a wide range of materials at the nanometer scale while adding capabilities lacking in passive tips [55,157,177]. Such active tips can selectively pattern features by addressing individual tips simultaneously, and can control the feature dimensions by adjusting the force, electrical potential, or temperature of the tip. This capability allows an array of such active tips to independently fabricate unique structures simultaneously. The integration of a height or position sensor into each active tip in an array would allow scalable and controlled nano-manufacturing through repeated fabrication and imaging of structures. In certain cases such as tips with integrated heater-thermometers, the same functionalization used for nano-manufacturing (integrated heater) also serves as a height sensor [178,179,180]. Recent advances in microfabrication, materials integration, and control systems and electronics have enabled the development of active tip arrays that are compatible with commercial AFMs, thereby showcasing the potential for scalable tip-based manufacturing for many industrial applications.

**Figure 12:**
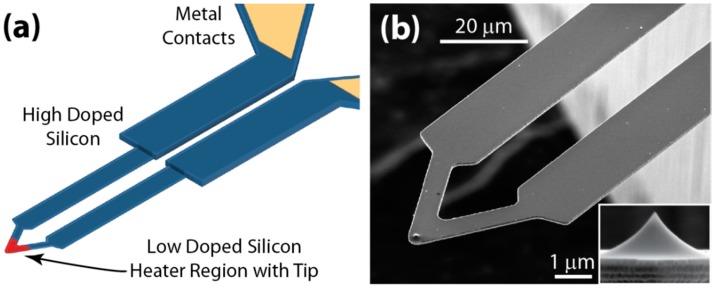
(**a**) Schematic and (**b**) SEM micrograph of a heat-able microcantilever. The cantilever has an integrated resistive heater near the cantilever tip (inset).

Tips with integrated heaters are one of the most widely used types of active tips for tip-based nanofabrication. Figure 12 shows a popular example of such heated cantilevers with integrated heaters that are made out of single-crystal doped silicon. The cantilever is U-shaped and made of differentially doped silicon such that the cantilever legs are high doped to carry current and the cantilever free-end is low doped to form a resistive heater. Passing current through the cantilever causes more than 90% of the power to be locally dissipated at the heater, and the heater temperature can reach temperatures over 1000 °C [181]. Several studies have investigated the heat transfer mechanisms within and from the cantilevers under various operating conditions and environments [55,182]. Such heated tips can pattern sub-20 nm silicon and metal structures. Thermal time constants of heated tips determine the switching speed for tip-based writing, and they range from a few µs to hundreds of µs. The size of fabricated features largely depends on the tip radius, which is in the order of tens of nanometers. The heated tips are used for thermal topography imaging for post-patterning metrology. Though these tips were originally developed for data-storage applications in the early 1990s [41], they currently underpin of a wide variety of nano-manufacturing techniques including mechanical or chemical modification of surfaces, deposition of materials onto surfaces, and heat-assisted materials synthesis [55]. In addition, these tips are currently used for thermal topography measurement, materials property analysis, and heat transfer measurement at the nanometer scale [55].

**Figure 13:**
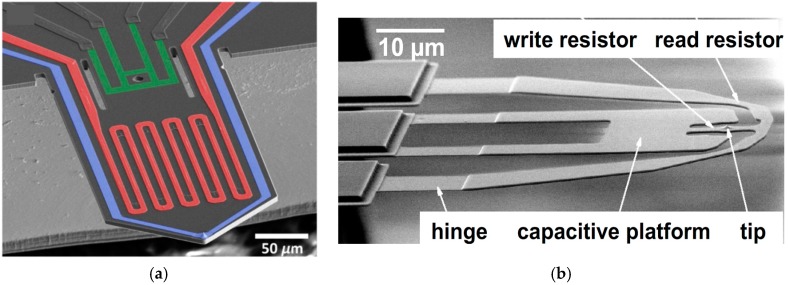
(**a**) SEM image of the cantilever with thermal bimorph actuation (red), piezoresistive sensor (green), and electrode to the conducting tip (blue). A cantilever is used for advanced electric-field scanning probe lithography on molecular resist [183]. (**b**) Heated cantilever with a capacitive platform for electrostatic actuation, heated tip, and resistive read sensor [155].

Active tips with self-actuation and sensing capabilities provide high operational flexibility and can also be operated independently in parallel for scalable tip-based nanofabrication. Figure 13a shows a silicon cantilever with a tip, a thermal bimorph actuator, and a piezoresistive deflection detection system [183]. Such cantilevers have been used for tip-based lithography on polymer resists by utilizing a spatially confined low-energy electron emission from the cantilever tip. Moreover, the same cantilever tip can be used for imaging of the substrate using an un-biased tip and the piezoresistive deflection sensors. Figure 13b shows the most recent class of doped silicon heated cantilevers developed by IBM, with an electrostatic actuation platform, a resistive read sensor, and a heatable cantilever tip [155]. These cantilevers are used for high-speed and high-resolution thermal scanning probe lithography (tSPL) [184].

**Figure 14:**
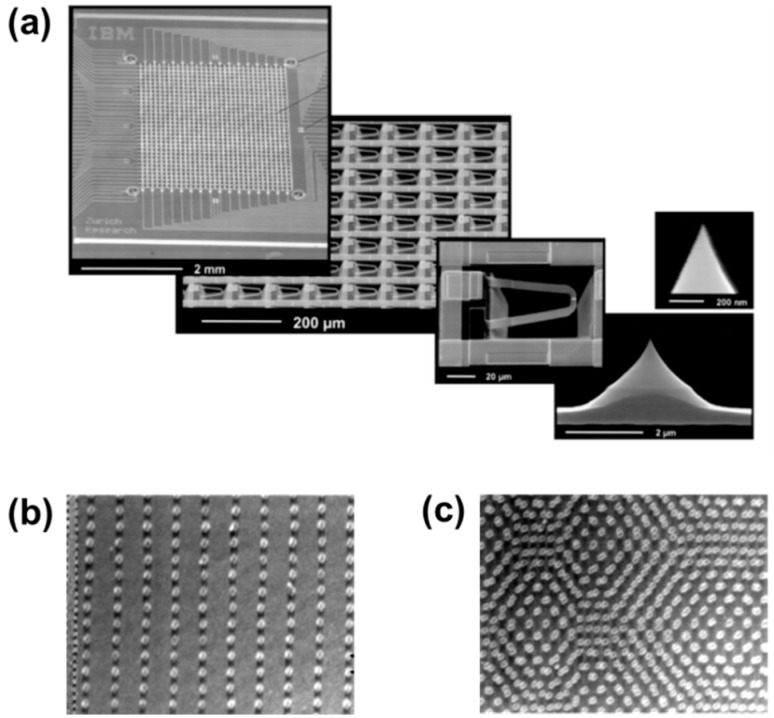
(**a**) SEM images of the IBM Millipede, which contained an array of 1024 cantilever tips, each with an integrated heater-thermometer above the tip [30]. (**b**,**c**) Data bits with aerial densities of up to 400 Gbit/in^2^ written on polymer thin-films using the Millipede [203].

The concept of active tip arrays was first demonstrated by the Quate group through parallel lithography using cantilevers with integrated piezoelectric actuators and piezoresistive height sensors [185,186]. Parallel and independent operation of active tips was subsequently pioneered by IBM during the development of “Millipede”, shown in Figure 14—a data storage system using as many as 1024 heatable tips. Self-heating within the tip caused the underlying polymer thin-film to soften and mechanical pressure applied to the tip resulted in an indent (or a binary 1) in the polymer film. Multiple parameters including the self-heating temperature and the heating rate, and the mechanical force determined the bit indent sizes, which could be as small as a few tens of nanometers. Data densities as high as 4 Tbit/in^2^ using a single tip, and data write rates of 10 Mbps using all tips were demonstrated [30,55,187].

There are several design and implementation challenges in the development of scalable nano-manufacturing systems. In order to manufacture the same or similar structures with each tip and precisely control the dimensions of the fabricated features, control systems are used to control parameters such as the tip force, tip bias, tip temperature, etc. Though these control systems can be implemented in software [188], autonomous implementation of the control system using integrated circuitry [180] or dedicated field gate programmable arrays [30,189,190] are more scalable with the number of active tips. As the tip array becomes large, additional control mechanisms are required to ensure that the tips are leveled relative to the sample, to apply uniform pressure onto the substrate, and to account for thermal expansion of the carrier chip [30], especially when the tips do not have independent force sensing and actuation mechanisms. Besides utilizing multiple active tips, scalable nano-manufacturing systems necessitate the integration of height sensors to image the fabricated features. Arrays could use separate tips for imaging and fabrication; however, this would increase the size of the array and introduce other challenges including alignment and co-registration of features between the imaging and fabrication tips. Alternatively, the arrays could use the same tip for imaging and manufacturing. The integration of multiple elements such as a height sensor, actuator, and functionalization for nano-manufacturing can significantly complicate the design, fabrication, and packaging of the array chip and each individual tip. Furthermore, the total number of electrical leads for each tip [30] and crosstalk [30,191] between the different elements on each tip scales with the number of elements such as sensors and actuators. Moreover, each topography sensor in the array necessitates dedicated high-speed data acquisition channels. The desire to fabricate ever-smaller features makes data acquisition and processing even more challenging and often necessitates significant signal processing electronics and custom instrumentation software [155,180,183].

**Figure 15:**
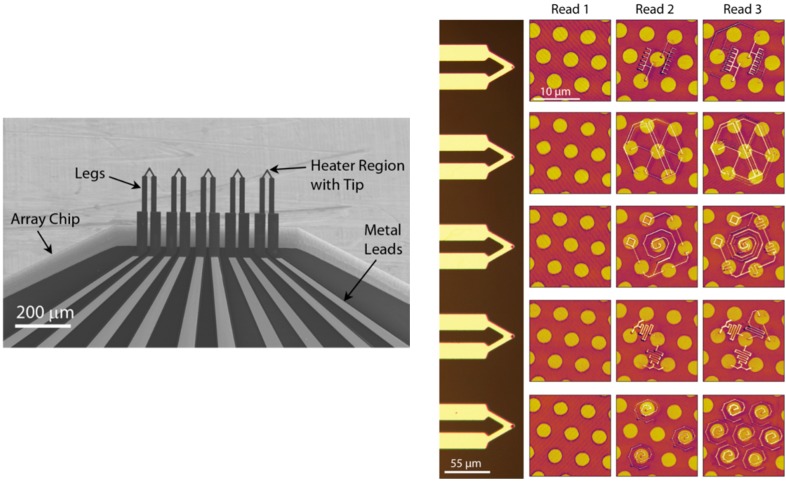
Parallel thermomechanical nanolithography using an array of 5 heated cantilevers. Read 1: thermal image of the substrate. Read 2: after first cycle of lithography. Read 3: another lithography cycle at lower writing temperature to pattern lower and narrower features [178].

The aforementioned complications in the array design sometimes necessitates the development of the SPM instrument specifically adapting to the array, thereby ballooning the cost and limiting the large-scale adoption of arrays in industry and academia. In some cases, the complex array technology has resulted in poor manufacturing or imaging performance, thereby defeating the purpose of arrays [192,193,194,195,196,197,198]. One popular solution has been to simplify the array system design wherever possible. In the case of the Millipede, the same integrated heaters in the heated tips also served as sensors to image the written features (read the written data). Recently, a group led by King developed arrays of 5 and 30 heated tips that were integrated into multiple commercially available AFMs with minimal custom hardware. Using these arrays they demonstrated parallel TCNL [199], parallel and independent thermomechanical lithography as shown in Figure 15 [178], and high speed, large-area topography imaging [178,200]. In addition, arrays with ultra-nanocrystalline diamond tips or with lower-stiffness cantilevers have been developed to reduce tip wear during the use of cantilever tips under extreme operating conditions, such as high loading force, high scan speed, and hard scanning substrate [201,202].

Conventional AFM tips can have individual addressing capabilities with external thermal, electrical, or optical sources. Radiative heating of AFM tips using femtosecond laser pulses focused on the tip is used to create nanocraters on thin metal films [103]. In addition, a controllable surface nanomachining of thin gold films with a feature size of about 10 nm has been demonstrated by utilizing the local field enhancement in the near-field of an AFM tip using the ultrashort pulsed-laser radiation [101]. Applying an electrical bias through a sharp tip and a conducting surface can induce a chemical reaction over a wide range of materials in both ambient and liquid environments. In addition, biased tips can guide and pattern the self-assembled monolayers, enabling tip-based electrochemical fabrication [89,204]. Besides AFM tips, an array of mold-fabricated PDMS tips are used as a conformal photomask in near-field photolithography using a halogen light source to create features as small as 30 nm [205].

## 5. Conclusions and Outlooks

Several technology challenges need to be solved in order for TBN to grow into a truly scalable nano-manufacturing technology.

First, an easy to implement approach of maintaining the desired distance between each individual tip to the substrate is needed. The conventional way of using a laser reflection and optical quadrupole as used in AFM systems only works for a single tip and is difficult to scale up to address each tip in an array form. New approaches that are capable of aligning the tip array with the specific distance to the substrate are in demand. For example, an individual sensor mounted on a microcantilever for measuring tip-substrate distance can be one solution for such applications as integrated thermometers or piezoresistors. In addition, substrates could be polished by chemical mechanical polishing (CMP) [206] to achieve smoother surfaces, which can make it easier for all tips to stay at an optimal working distance. From an instrumentation perspective, an interferometer system [207] can also be implemented into the TBN system to help adjust the plane of the sample stage to be parallel to the plane of the tip arrays with a nanometer-scale resolution.

Second, a way of integration with semiconductor manufacturing method is required. This can be achieved by patterning resist materials such as PMMA for etch masks in subsequent etching steps. Third, a way of producing desired tip sharpness reliably is required to ensure feature uniformity produced by TBN, as the feature size is highly dependent on the tip sharpness. Zhou’s group has demonstrated an approach of batch-fabricating microcantilevers with a controlled tip radius [208]. Fourth, as the numbers of tips increase, the electronic circuit and computers handling the signal for each tip also need to be upgraded to be faster and more powerful.

Current TBN methods can also borrow technology advances from fields in electron beam lithography and focused ion beams to boost throughput and speed. For example, Duan et al. recently demonstrated a “sketch and peel” strategy to enhance the speed of electron-beam lithography [209] and focused ion beam milling by only patterning the pre-sketched outlines [210], and the same concept might also work for TBN.

In terms of application, early TBN application is mostly focused on electronic device and data storage application since they originate in STM and AFM. With the advancement of tip fabrication in the level of millions and a greater choice of materials in TBN, we will see more frequent use of TBN in surface patterning for functional nanostructured surfaces, chemistry synthesis, and biomaterials patterning.

## Figures and Tables

**Table 1:** Summary of different tip-based nanofabrication (TBN) techniques. tDPN: thermal dip pen nanolithography; DPN: dip pen nanolithography; TCNL: thermal-chemical nanolithography.

TBN Types	Reported Resolution	Scanning Speed	Material Choice	Need Vacuum	Need Humidity Control	Advantages	Disadvantages
Atom-removal-based	Sub-1 nm [152]	Slow 80 nm/s [40]	Semiconductors metals	Ultrahigh vacuum (<10^−9^ Torr)	No	Atomic precision, good for building molecular devices	Super slow, low throughput
DPN	10 nm [153]	Slow 0.1–4 µm/s [105,154]	Biological Materials, Chemicals	No	yes	Good for biological patterning, Compatible with self-assembly	Slow speed, need inking
tDPN	10 nm [59]	Medium 0.1–200 µm/s [59,63]	Polymer & metals with low melting temperatures	No	No	Compatible with semiconductor processing, good reproducibility	Medium speed, need inking
Thermal-mechanical	Sub-20 nm [77]	Super-fast 20 mm/s [155]–1.25 m/s [156]	Polymer	No	No	Super-fast, left indentation	Need extra processing to obtain usable nanostructures
TCNL	10 nm [157]	Fast 1 mm/s [157]	Specific Resist	No	No	Fast, grey scale chemical patterning	Need specific polymer resist and require heated AFM tips
Mechanical removal	10 nm [158]	Medium 0.1–40 µm/s [122]	Metal, Semiconductors, Graphene	No	No	Easy to implement, wide selection of materials	Tip wear, debris formation, speed needs to be tuned to the material properties
Electro-chemical	4 nm [159]	Fast 0.5 µm/s–1 mm/s [157]	Metal, Semiconductors, Graphene	No	Yes	Fast, Room Temperature	Need electrical bias, Limited oxide thickness, Limited processing speed
Optical	10 nm [99]	Medium 1–20 µm/s [103]	Metal, polymer	No	No	Easier to scale up, ambient conditions	Requires extra optics
Field Emission	35 nm [149]	Fast 2 µm/s [151]–1 mm/s [150]	Resist, spin-on glass	Yes/No	No	No proximity effect compared to high energy electron beams	Requires electron-sensitive resist, external circuits to control the small current
